# Resection and Reproduction of the Maxillae*Abstract of paper read before the American Medical Association of Phila delphia, June, 1897.

**Published:** 1897-08

**Authors:** G. Lennox Curtis

**Affiliations:** New York. Formerly Professor of Oral and Facial Surgery, New York Dental School; And Instructor in the New York Post Graduate Medical School and Hospital


### Resection and Reproduction of the Maxillae.*

BY G. LENNOX CURTIS, M. D., NEW YORK.

Formerly Professor of Oral and Facial Surgery, New York Dental School; and Instructor in the New York Post Graduate Medical School and Hospital.

The purpose of this paper is to show the profession the importance of special study and instruction in oral and facial diseases and that these are worthy of the same consideration as is given to any of the fully recognized specialties in medicine. Until they do appear in the curriculum of the medical school, the faculty will not have done its duty to the student. The field covered by the general surgeon is altogether too great for a careful consideration of any part where such minuteness is required to save and assist nature in doing her work. The surgeon most capable of successful teaching in this line is he who has been a thorough and conservative dentist. Like produces like—this applies to every department in nature. The periosteum, under favorable conditions, will reproduce the substance it covered. That of the ramus of the jaw will only make the thin lamina of bone which nature has originally placed there, while that of the molar and the body of the jaw reproduces a dense structure differing materially in texture.

If the functions of a part be permanently lost, reproduction is not a necessity, nature supplying only that part which is required. If the teeth have been extracted with a view of remaining out, the portion of the jaw which is required to nourish them is not reproduced, but where the teeth are replaced and retained, all or sufficient of the bone is reproduced and re-attaches them to the jaw—such I have seen in active use for years.

The only condition I can ascribe for the removal of the periosteum, is where it is attacked by disease, such as cancer, and the entire structure destroyed. When the bone alone is destroyed, as in necrosis, cystic tumors, or from pressure by resistance of growth, as a tumor of the antrum, I see not the

*Abstract of paper read before the American Medical Association of Philadelphia, June, 1897.

slightest necessity for removing this natuıal sheath, but on the contrary, every reason for retaining it. I have seen Billroth, Van Burgmann, Agnew, Ashurst, Garretson, and other great surgeons resect the jaw, but they invariably employed Augfelder's, Ferguson's or Langenbeck's method, except in cases of necrosis, where but small sections were involved. Liston, Tait, Barton, Mutter and Gross also followed on these lines.

But what can we say for the subject? Partially or wholly jawless, maimed and disfigured for life, a repulsive and pitiable object to others and a shrinking annoyance to himself. Is it not time to call a halt and look this matter squarely in the face?

I do not censure the surgeon whose opportunities to acquire knowledge have been dwarfed by the oversight of his teachers.

My method to obtain the best results in the preservation of the contour of the jaw is by retaining the necrosed bone in position until the periosteum has been so strengthened by the reproduction as to allow nature's outlines to be maintained, employing it as an inter-osseous splint.

Where it is necessary to remove the bone, I retain the contour of the face by gauze packing, and change from time to time till the bone is sufficiently reproduced to resume its shape. This requires frequent dressing so that the amount of pus may be kept at a minimum. The teeth are retained in position by means of inter-dental splints or ligatures. Where the teeth are lost, I place other teeth in the opening when the wound is nearly closed, maintaining them by artificial support, and allowing the bone to form around them. Where the destruction of the bone has been great, and the periosteum too weak to retain the jaw in position during the process of reproduction, I use an inter-dental splint as employed by Liston over fifty years age, in which the upper and lower teeth properly occlude. By hastening slowly, the danger of wounding the dental nerve is materially lessened.

Scarce can we read a text-book in which is not found methods of treatment of facial disease in vogue half a century ago.

In 1886 I operated on a young woman, aged twenty-three, with the following history: Four years previous, after suffering much pain in the face, which was swollen, a fistula appeared in the lower jaw, which was diagnosed as being from an abcessed tooth. The gums around the teeth were swollen and inflamed, the molars and the second bicuspids were extracted and the pus continued to flow. Her health rapidly diminished, menses ceased and had not returned although constantly under medical and surgical treatment. Examination revealed the emaciated condition of the patient. She was suffering from blood-poisoning, was highly nervous and hysterical, had no desire for food and had lost the sympathy of her doctors and family.

In the left inferior maxilla where the tooth had been extracted, there were granulations. A boggy condition of the mucous membrane extended all along the side of the jaw. Over the ramus it was particularly inflamed. The probe readily passed beneath the periosteum, and far up along the ramus.

The patient was then too sick for an operation with a view to best results. The wound was cleansed daily to lessen the amount of pus, and for one month the patient was placed under most rigid restorative treatment with good results. I found that under the local stimulating treatment, bone had been sufficiently reproduced to strengthen the periosteum, so that when I removed the dead jaw the contour was preserved. The cause of the trouble I found to be a wisdom tooth lying transversely at the neck of the jaw, immediately under the condyle. This along with the granulation and debris was removed. The wound was packed continually until healthy granulations filled in the periosteum; the jaw, minus the teeth, was reproduced with all its usefulness. Complete restoration to health and a gain of twenty pounds in weight followed this work. The nerves and vessels in the jaw were not injured, and no paralysis resulted.

Before my class at the New York Post Graduate Medical School and Hospital, on March 25, 1893, I operated on a lad, fifteen years of age, who gave the following history:

Three years before, while at play, he ran against a lamp post, striking the left side of his face and bruising it severely. A year later there appeared on the face, over the molar bone, a hard lump, which continued to increase until it was the size a hen's egg, preventing the boy from seeing with that eye objects on the ground near by, without bending his head. He had not realized any special pain or discomfort from the tumor. Thinking the trouble arose from the abcessed teeth, his dentist extracted the upper left first bicuspid which showed no evidence of being diseased. I diagnosed an osseous tumor of the antrum, and found that the molar and superior maxillary bones were completely destroyed by the direct pressure against them, only the periosteum remaining. Not only was the tumor directed outward but downward, depressing the roof of the mouth and extending beyond the alveolar process against the buccinator muscle. An incision was made through the periosteum encircling the teeth, as seen in the specimen here presented, in which the tumor and teeth are attached, and it will be noticed that only a small part of the alveolar process remained intact. This with the teeth was removed, the entire side of the face falling into the cavity made by their absence, so completely were the molar and superior maxillary bones destroyed. The inferior orbital ridge and zygoma only resisting the pressure of the tumor. A profuse hemorrhage followed its removal, but was rapidly checked with hot water. The wound was packed with aristol and gauze, and the contour of the face secured, the periosteum united with the sutures. Through this operation the wound was dressed until the shape of the face was permanently restored. Time of operation, twenty-five minutes.

The following day there was considerable œdema which readily subsided. From day to day the dressing was changed until the periosteum could support itself, and in two weeks the case was dismissed from the hospital.

The antrum was douched daily until restoration was complete. An artificial denture was made to replace those lost, to give the normal fullness to the mouth. In this operation there was no external wound, consequently no necessity for ligature and no scarring of the face which would necessarily follow had the operation been done on the lines drawn in general surgery. The wound completely healed in six weeks with no deformity of the face. I have seen the case from time to time and in every way it is eminently satisfactory.

To facilitate cleansing of the wound and destruction of the pus, let me reccommend to you electrozone, the best of all agents I have found for this purpose. Under its use the pus melts away like due before the morning sun.

I have done many cases similar to these stated and without deformity in any instance. All of them are accompanied by blood poisoning and have usually been treated for rheumatism, malaria and typhoid fever for months and even years, before the error is discovered.

This conservative method is not condusive to a fine selection of pathological specimens, as a recovery of a part without blemish leaves only the history of the case and the statement of the patient as proof of the malady.

The student should have a chance to see in practice the methods he wishes to adopt. In this city, only recently, at our great University no end of strife resulted from a determined and successful effort of the dental department to make a place in the hospital for their oral surgeon, with equal right to operate. This was the beginning of the end when all such institutions must of necessity adopt the same course. Why confine this work to so-called major operations, when they can be simplified to the minor class by annexing such men, skilled in dentistry and medicine alike, to the medical faculty!

Let us hope that the controllers of such faculties will appreciate that "knowledge is power" and that new methods are a necessity.

